# Application note: TDbasedUFE and TDbasedUFEadv: bioconductor packages to perform tensor decomposition based unsupervised feature extraction

**DOI:** 10.3389/frai.2023.1237542

**Published:** 2023-09-01

**Authors:** Y-h. Taguchi, Turki Turki

**Affiliations:** ^1^Department of Physics, Chuo University, Tokyo, Japan; ^2^Department of Computer Sciences, King Abdulaziz University, Jeddah, Saudi Arabia

**Keywords:** tensor decomposition, feature selection, unsupervised learning, gene expression, multiomics

## Abstract

**Motivation:**

Tensor decomposition (TD)-based unsupervised feature extraction (FE) has proven effective for a wide range of bioinformatics applications ranging from biomarker identification to the identification of disease-causing genes and drug repositioning. However, TD-based unsupervised FE failed to gain widespread acceptance due to the lack of user-friendly tools for non-experts.

**Results:**

We developed two bioconductor packages—TDbasedUFE and TDbasedUFEadv—that enable researchers unfamiliar with TD to utilize TD-based unsupervised FE. The packages facilitate the identification of differentially expressed genes and multiomics analysis. TDbasedUFE was found to outperform two state-of-the-art methods, such as DESeq2 and DIABLO.

**Availability and implementation:**

TDbasedUFE and TDbasedUFEadv are freely available as R/Bioconductor packages, which can be accessed at https://bioconductor.org/packages/TDbasedUFE and https://bioconductor.org/packages/TDbasedUFEadv, respectively.

## 1. Introduction

Tensor decomposition (TD)-based unsupervised feature extraction (FE) has been successfully applied to a wide range of problems (Taguchi, 2020) since it was introduced several years ago (Taguchi, 2017). Despite its success, the method failed to gain widespread acceptance, possibly due to the lack of practical tools to perform TD. To address this end, we have developed two bioconductor packages, TDbasedUFE and TDbasedUFEadv, which allow researchers to perform TD-based unsupervised FE easily without the need of detailed knowledge of TD. The purpose of this manuscript is not to demonstrate the superiority over the other methods, since the superiority over the other methods has already been demonstrated in numerous studies cited below. The purpose of this manuscript is to simply inform about the implementation of the established method into easy-to-use environment.

## 2. Methods

TD-based unsupervised FE (Taguchi, 2017) was derived from principal component analysis (PCA)-based unsupervised FE (Taguchi and Murakami, 2013), which was introduced 10 years ago. As datasets grew in complexity and began to include multiple measurement conditions, such as comparisons of multiple tissues from human subjects rather than just those from human patients restricted to a single tissue, tensors were employed instead of matrices. Tensors, which can have multiple indices, each of which can have multiple comparison criteria, better accomodate complex data structures. For example, a three mode tensor *x*_*ijk*_ can naturally store the expression of *i*th gene at *k*th tissue of *j*th human subjects. In contrast, matrices with only two indices corresponding to rows and columns require combining the tissue index and the human index into a single column, rendering data interpretation challenging.

TDbasedUFE and TDbasedUFEadv are user-friendly packages that allow individuals who are unfamiliar with tensors to perform unsupervised feature extraction. Since a matrix can be considered as a two-mode tensor, these packages can also be used to apply PCA-based unsupervised FE to the dataset. TDbasedUFE focuses on two popular functions developed for TD-based unsupervised FE, including the identification of differentially expressed genes (DEGs) and multiomics analyses. For the DEG identification, the basic algorithm is based on a recent study (Taguchi and Turki, 2022b) that established a new standard deviation (SD) optimization approach. For multiomics analysis, the basic algorithm is based on the same study (Taguchi and Turki, 2022c). However, TDbasedUFE also incorporates SD optimization, which was not available when the study was published. Although the algorithm is not specifically designed for DNA methylation profiles, we found that the approach described in the study (Taguchi and Turki, 2022b) is also applicable to DNA methylation profiles (Taguchi and Turki, 2023). In this regard, any type of differential analysis on single omics data can be performed by functions implemented in TDbasedUFE. In fact, we have shown (Turki et al., 2023) that histone modification profiles can be analyzed using the algorithm described in the study (Taguchi and Turki, 2022b).

**Figure 1:**
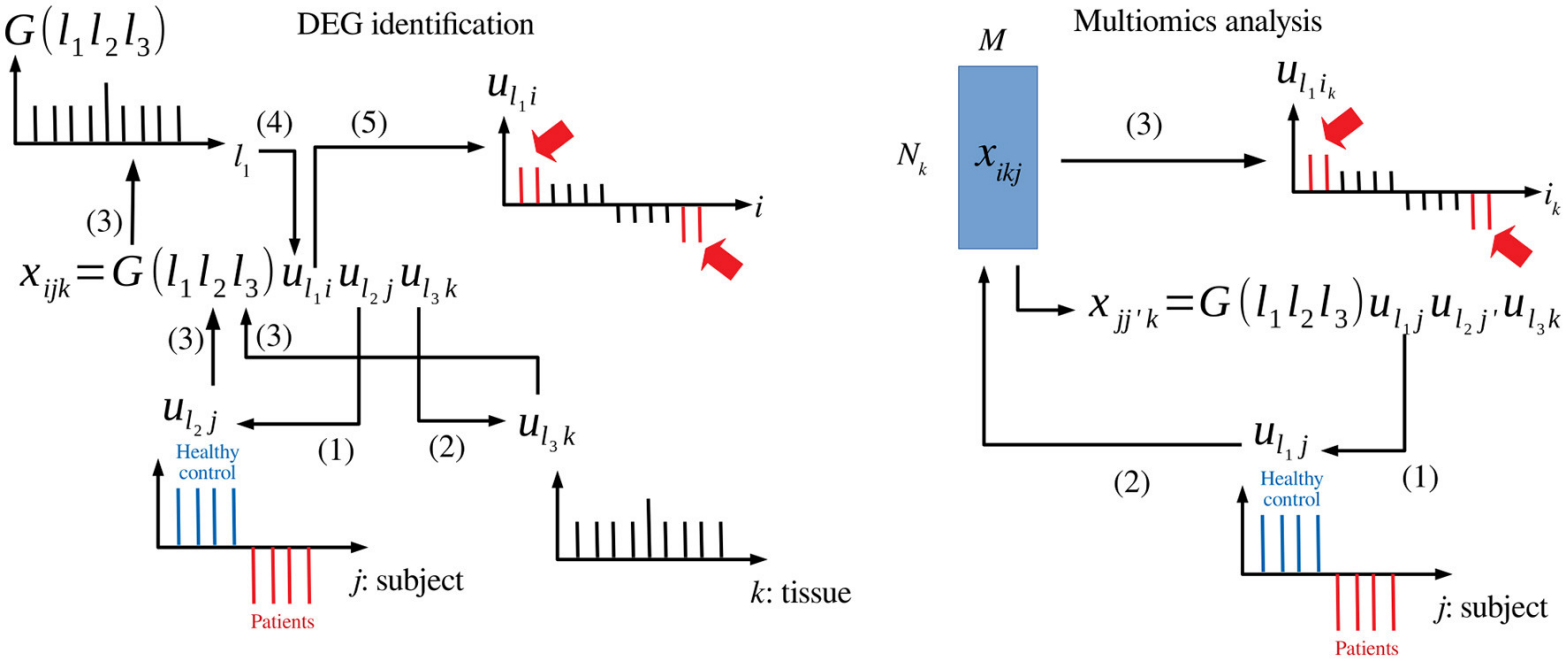
Schematic diagram that explains TD-based unsupervised FE. **Left**: DEG identification, (1) *u*_ℓ_2_*j*_ associated with the distinction between patients and healthy controls is selected. (2) *u*_ℓ_3_*k*_ associated with tissue specificity is selected. (3) *G*(ℓ_1_ℓ_2_ℓ_3_) is investigated with fixed ℓ_2_ and ℓ_3_. (4) *u*_ℓ_1_*i*_ with *G* of the largest absolute value is selected. (5) *i*s (indicated in red) whose absolute values are significantly larger than expected are selected. **Right**: Multiomics analysis, (1) *u*_ℓ_1_*j*_ associated with the distinction between patients and healthy controls is selected. (2) *u*_ℓ_1_*i*_ is computed from *u*_ℓ_1_*j*_. (3) *i*s (indicated in red) whose absolute values are significantly larger than expected are selected.

TDbasedUFE and TDbasedUFEadv accept a multiple omics profile dataset formatted as a tensor. TD is applied on this dataset using Tucker decomposition based on higher order singular value decomposition (HOSVD) (Taguchi, 2020) algorithm. For instance, if $x_{ijk}\in {\mathbb{R}}^{N\times M\times K}$ represents the gene expression of *i*th gene of *j*th human subject's *k*th tissue (Figure 1 left), TD is applied to *x*_*ijk*_, and the following equation is obtained:


(1) $$\begin{array}{l} x_{ijk}=\overset{N}{\underset{\ell_{1}=1}{\sum }}\overset{M}{\underset{\ell_{2}=1}{\sum }}\overset{K}{\underset{\ell_{3}=1}{\sum }}G\left(\ell_{1}\ell_{2}\ell_{3}\right)u_{\ell_{1}i}u_{\ell_{2}j}u_{\ell_{3}k} \end{array}$$

where *G* ∈ ℝ^*N*×*M*×*K*^ is a core tensor that represents the weight of the product *u*_ℓ_1_*i*_*u*_ℓ_2_*j*_*u*_ℓ_3_*k*_ to *x*_*ijk*_, and $u_{\ell_{1}i}\in {\mathbb{R}}^{N\times N}$, $u_{\ell_{2}j}\in {\mathbb{R}}^{M\times M}$, and $u_{\ell_{3}k}\in {\mathbb{R}}^{K\times K}$ are singular value matrices and orthogonal matrices. Initially, singular value vectors attributed to samples, *u*_ℓ_2_*j*_ and *u*_ℓ_3_*k*_, are investigated to identify those of interest. For instance, *u*_ℓ_2_*j*_ represents the distinction between healthy controls and patients, and *u*_ℓ_3_*k*_ represents tissue specificity (e.g., expressed only in the heart). Then, the singular value vectors attributed to genes (i.e., features) *u*_ℓ_1_*i*_ that share *G* of the largest absolute value with the identified *u*_ℓ_2_*j*_ and *u*_ℓ_3_*k*_ are selected. Features (*i*s) with larger absolute values of *u*_ℓ_1_*i*_ are identified based on *P*-values computed by assuming that *u*_ℓ_1_*i*_ obeys a Gaussian distribution (null hypothesis) as follows:


(2) $$\begin{array}{l} P_{i}=P_{\chi^{2}}\left[>{\left(\frac{u_{\ell_{1}i}}{\sigma_{\ell_{1}}}\right)}^{2}\right] \end{array}$$

where $P_{\chi^{2}}\left[>x\right]$ is the cumulative χ^2^ distribution where the argument is larger than *x*, and σ_ℓ_1__ is the optimized standard deviation such that *u*_ℓ_1_*i*_ obeys Gaussian distribution as much as possible (see Taguchi and Turki, 2022b for more details about how to optimize σ_ℓ_1__). Then *P*_*i*_s are, then, adjusted using the Benjamini–Hochberg criterion to consider multiple comparison correction. Finally, *i*s with adjusted *P*_*i*_ less than threshold value (typically, 0.01) are selected.

When TDbasedUFE is applied to multiomics datasets (Figure 1 right), the multiomics profiles are formatted as $x_{i_{k}j}\in {\mathbb{R}}^{N_{k}\times M}$ (i.e., *k*th omics datasets are associated with as many as *N*_*k*_ features). The *x*_*i*_*k*_*j*_s are multiplied with each other to obtain the following equation:


(3) $$\begin{array}{l} x_{jj^{\prime }k}=\overset{N_{k}}{\underset{i_{k}=1}{\sum }}x_{i_{k}j}x_{i_{k}j^{\prime }}\in {\mathbb{R}}^{M\times M\times K} \end{array}$$

HOSVD is, then, applied to $x_{jj^{\prime }k}$ as follows:


(4) $$\begin{array}{l} x_{jj^{\prime }k}=\overset{M}{\underset{\ell_{1}=1}{\sum }}\overset{M}{\underset{\ell_{2}=1}{\sum }}\overset{K}{\underset{\ell_{3}=1}{\sum }}G\left(\ell_{1}\ell_{2}\ell_{3}\right)u_{\ell_{1}j}u_{\ell_{2}j^{\prime }}u_{\ell_{3}k}. \end{array}$$

After identifying *u*_ℓ_2_*j*_ coincident with labels (e.g., patients and healthy control), singular value vectors attributed to individual features associated with *k*th omics are computed as follows:


(5) $$\begin{array}{l} u_{\ell_{1}i_{k}}=\overset{M}{\underset{j=1}{\sum }}u_{\ell_{1}j}x_{i_{k}j}\in {\mathbb{R}}^{N_{k}} \end{array}$$

Moreover, *P*_*i*_*k*__ is, then, computed as follows:


(6) $$\begin{array}{l} P_{i_{k}}=P_{\chi^{2}}\left[>{\left(\frac{u_{\ell_{1}i_{k}}}{\sigma_{\ell_{1}}}\right)}^{2}\right] \end{array}$$

and *i*_*k*_s associated with adjusted *P*_*i*_*k*__ less than 0.01 are selected.

In contrast to TDbasedUFE, which can perform only two tasks, TDbasedUFEadv can perform more complicated tasks. For example, TDbasedUFEadv can perform (Ng and Taguchi, 2020) integrated analysis of two omics profiles that share samples and reduce the memory required by summing up the sample index. TDbasedUFEadv can also perform integrated analysis of two omics profiles that share features (Taguchi and Turki, 2019). TDbasedUFEadv can also perform integrated analysis of multiple (more than two) omics profiles that shared features (Taguchi and Turki, 2022a) or samples (Taguchi and Turki, 2021).

## 3. Results

The full list of identified features, as well as the results of the enrichment analysis in this section, is presented in Supplementary material. For further details, please also refer to the Supplementary Document.

Numerous applications of TD-based FE were proposed since the publication of our book (Taguchi, 2020). Here, we present a few examples to demonstrate the usefulness of TDbasedUFE based on the ACC.rnaseq data from RTCGA.rneseq (Kosinski, 2023) package in Bioconductor. The labels used to select singular value vectors attributed to samples were patient.stage_event.pathologic_stage composed of four classes (“stage i” to “stage iv”). A tensor $x_{ijk}\in {\mathbb{R}}^{N\times 9\times 4}$ represents the expression of *i*th gene of *j*th replicates of *k*th stage. HOSVD was applied to *x*_*ijk*_, and we obtained TD, as shown in Equation (1) (please refer to the Supplementary Document for the R code to perform DEG identification using TDbasedUFE). Since *u*_ℓ_2_*j*_ is attributed to replicates, *u*_ℓ_2_*j*_ is expected to have constant values, regardless of how *j* and ℓ_2_ = 1 turned out to satisfy this requirement (Supplementary Figure S1 left). On the other hand, *u*_ℓ_3_*k*_ is expected to have monotonic dependence on *k* (Supplementary Figure S1 right); and we found that ℓ_3_ = 3 was most coincident with monotonic dependence on *k*. Once ℓ_2_ and ℓ_3_ are selected by the user with the interactive interface, TDbasedUFE automatically selects *u*_ℓ_1_*i*_ with which *i*s are selected. As a result, 1,692 genes were selected with the threshold-adjusted *P*-value of 0.01.

To evaluate the ability of TDbasedUFE to select genes, we applied DESeq2 (Love et al., 2014), a state-of-the-art method, on *x*_*ijk*_. DESeq2 is not applied to *x*_*ijk*_ but to the unfolded matrix $x_{i\left(jk\right)}\in {\mathbb{R}}^{N\times 36}$ where *j* and *k* are merged into a column index (see the Supplementary Document for the R code to perform DEG identification using DESeq2). We identified as few as 138 genes associated with adjusted *P*-values less than 0.01 using DESeq2. Thus, from the perspective of the number of identified DEGs, TDbasedUFE is clearly superior to DESeq2.

However, identifying a higher number of DEGs does not necessarily mean that all of the identified DEGs are biologically relevant. To evaluate the biological relevance of the DEGs selected by TDbasedUFE, we used the enrichR (Jawaid, 2023) package in CRAN, as demonstrated in the vignette “Enrichment” in the TDbasedUFEadv package considering the “KEGG 2021 HUMAN,” “GO Molecular Function 2015,” “GO Cellular Component 2015,” and “GO Biological Process 2015” categories. When 1,692 genes selected by TDbasedUFE are considered, 129, 151, 143, and 923 terms were found to be associated with adjusted *P*-values less than 0.05 for the “KEGG 2021 HUMAN,” “GO Molecular Function 2015,” “GO Cellular Component 2015,” and “GO Biological Process 2015” categories, respectively. On the other hand, when 138 genes selected by DESeq2 are considered, 0, 0, 3, and 12 terms are associated with adjusted *P* < 0.05 for the same categories. Thus, in terms of the number of biologically relevant terms identified, TDbasedUFE outperforms DESeq2.

To demonstrate the capabilities of TDbasedUFE on a multiomics dataset, we used the curatedTCGA (Ramos et al., 2020) package to retrieve profiles other than the gene expression of the ACC dataset in TCGA (please refer to the Supplementary Document for the R code to perform DEG identification using TDbasedUFE). We have collected miRNA ($x_{i_{1}j}\in {\mathbb{R}}^{1046\times 79}$), gene expression ($x_{i_{2}j}\in {\mathbb{R}}^{120501\times 79}$), and methylation data($x_{i_{3}j}\in {\mathbb{R}}^{48577\times 79}$) from curatedTCGA and applied TDbasedUFE on these data. After applying HOSVD to the generated tensor $x_{jj^{\prime }k}\in {\mathbb{R}}^{79\times 79\times 3}$, we found that *u*_7*j*_ (Supplementary Figure S2 upper) is associated with the distinction between four stages, and *u*_1*k*_ (Supplementary Figure S2 lower) is constant regardless of *k* (i.e., omics). *P*_*i*_*k*__ is attributed to *i*_*k*_ by Equation (6) using *u*_7*i*_*k*__ generated from *u*_7*j*_ by Equation (5). After correcting *P*_*i*_*k*__, we found that 23 out of 1,046 miRNAs, 1,016 out of 20,501 mRNAs, and 7,295 out of 485,577 methylation probes are associated with adjusted *P*_*i*_*k*__ less than 0.01 (these features are expected to be distinct between the four stages as well).

To compare the performence of TDbasedUFE with those of SOTA methods, we employed DIABLO, which is implemented in the mixomics package (Rohart et al., 2017) in Bioconductor (please refer to the Supplementary Document for the R code to perform mulitiomics analysis using DIABLO). Even we used the minimum setup (folds=2, nrepeat=1), DIABLO failed to converge to a solution within 3 h. When the recommended setup in the vignette (folds=10, nrepeat=10) was employed, DIABLO did not converge to the solution with few enough errors up to 10 components (ncomp=10) and showed no tendency for errors to decrease as the number of components increased (Supplementary Figure S3). As a result, we were unable to select features using DIABLO and had to conclude that TDbasedUFE outperformed DIABLO for this multiomics dataset.

To evaluate the biological relevance of miRNAs, mRNAs, and methylation probes identified by TDbasedUFE, we have uploaded these to various databases. First, we uploaded the identified miRNAs to DIANA-mirpath v3.0 (Vlachos et al., 2015) and found that many cancer-related KEGG pathways are enriched (please refer to the Supplementary Document for URL to DIANA-mirpath using these miRNAs). Next, we uploaded the identified mRNAs to Enrichr (Xie et al., 2021) and found many cancer-related pathways in the “KEGG 2021 Human” categories and various cancer cell lines. Finally, we uploaded 2,668 unique gene symbols associated with the identified 7,295 probes to Enrichr and found several cancer-related pathways in “KEGG 2021 Human” and various cancer cell lines. In conclusion, the miRNAs, mRNAs, and methylation probes identified by TDbasedUFE are biologically relevant.

## 4. Discussion

Here, we have introduced TDbasedUFE and TDbasedUFEadv, two packages that can perform TD-based unsupervised FE without requiring extensive knowledge of tensor decompositions. Our results demonstrated that these packages outperform two SOTA methods, DESeq2 and DIABLO, when applied for DEG identification and multiomics analysis, respectively. With TDbasedUFE and TDbasedUFEadv, users can perform TD-based unsupervised FE easily and effectively.

In this implementation, TDbasedUFE/TDbasedUFEadv can accept variety of datasets generated from high throughput sequencing and/or old-fashioned microarray seamlessly. TDbasedUFE/TDbasedUFEadv can also accept the various combinations of these profiles as inputs (multiomics analysis). TDbasedUFE/TDbasedUFEadv can output the list of features associated with (adjusted) *P*-values. The possible output features are dependent on the input features. When genes are input, the output features are also genes. When genomic regions are input, the output features are also genomic regions. The list of features can be analyzed with enrichment analysis to understand biological meanings within the downstream analyses.

Current implementation does not have specific limitation since the implemented methods have already been tested over various topics in the numerous previous publications cited in this study. There are no future directions since it is a report to inform the implementation of established method.

As for other unsupervised gene selection methods, readers might check the review article Ang et al. (2016), although it listed as small as fifteen studies ranging from 2006 to 2012, which is relatively small compared with the number of our publications cited in this paper.

## Data availability statement

Publicly available datasets were analyzed in this study. This data can be found here: https://doi.org/10.18129/B9.bioc.TDbasedUFE; https://doi.org/10.18129/B9.bioc.TDbasedUFEadv.

## Author contributions

Y-hT and TT wrote an original manuscript, reviewed the manuscript, and validated the results. Y-hT has developed the package and performed analysis. All authors contributed to the article and approved the submitted version.
